# Intensive care unit capacity and mortality in older adults: a three nations retrospective observational cohort study

**DOI:** 10.1186/s13613-022-00994-x

**Published:** 2022-03-04

**Authors:** Ran Abuhasira, Matthew Anstey, Victor Novack, Somnath Bose, Daniel Talmor, Lior Fuchs

**Affiliations:** 1 Clinical Research Center, Soroka University Medical Center, Beer Sheva, Israel; 2 Faculty of Health Sciences, Ben-Gurion University of the Negev, Beer Sheva, Israel; 3 Sir Charles Gairdner Hospital, Perth, Australia; 4 School of Public Health, Curtin University, Perth, Australia; 5 School of Medicine, University of Western Australia, Perth, Australia; 6 Department of Anesthesia, Critical Care and Pain Medicine, Harvard Medical School, Beth Israel Deaconess Medical Center, Boston, MA USA; 7 Center for Anesthesia Research Excellence (CARE), Department of Anesthesia, Critical Care and Pain Medicine, Beth Israel Deaconess Medical Center, Boston, MA USA; 8 Medical Intensive Care Unit, Faculty of Health Sciences, Soroka University Medical Center, Ben-Gurion University of the Negev, Beer Sheva, Israel

**Keywords:** Intensive care unit, Aged, Elderly, Hospital mortality

## Abstract

**Background:**

Intensive care unit (ICU) admissions among older adults are expected to increase, while the benefit remains uncertain. The availability of ICU beds varies between hospitals and between countries and is an important factor in the decision to admit older adults in the ICU. We aimed to assess if a non-restrictive approach to ICU older adults admission is associated with a corresponding change in survival.

**Methods:**

Retrospective cohort study that included patients ≥ 80 years who were admitted to each of the three participating hospitals in Australia, Israel, and the United States (USA), between the years 2006–2015, each with distinct ICU capacities and admission criteria. The primary outcomes were in-hospital mortality and all-cause mortality at 6, 12, 18, and 24 months following index hospitalization.

**Results:**

The cohort included 62,866 patients with a mean age of 85.9 ± 4.6 years and 58.8% were women. The ICU admission rates were 22.5%, 2.6% and 2.3% in USA, Australia, and Israel, respectively. We constructed a model for ICU admissions based on the USA cohort (highest availability of ICU beds) and then calculated the expected probabilities for the Israeli and Australian cohorts. For the patients in the highest quintile of the admission model, actual ICU admission rates were 67.6% in USA, 22.1% in Australia and 6.0% in Israel. Of these, in-hospital death rates were 52.3% in Israel, 29.8% in Australia, and 22.1% in USA. Two years after hospital discharge, the survival rates in the USA and Australia were 53%, while in Israel 48%**.**

**Conclusion:**

ICU admission of adults ≥ 80 years is associated with increased in-hospital survival compared to ward admission, but survival rates 2 years later are similar.

**Supplementary Information:**

The online version contains supplementary material available at 10.1186/s13613-022-00994-x.

## Background

The number of older adults requiring intensive care unit (ICU) admission is increasing and is associated with significant costs and resource utilization [1, 2]. This trend is expected to increase given the ageing global population [3]. Age is a significant independent risk factor for in-ICU mortality, but uncertainty remains whether ICU admission in this population confers any short-term, or long-term benefit to patients [4–7].

This raises the question: should age be included in the decision-making processes for admission to intensive care, especially when there are capacity restraints [7]? A national French study showed that emergency and intensive care unit physicians were highly reluctant to consider intensive care unit admission of patients aged 80 years, despite the presence of criteria indicating that intensive care unit admission was certainly or possibly appropriate [8].

Lower ratio of ICU to non-ICU beds and association to higher mortality of older adults were described previously [9, 10]. Increasing ICU capacity led to an increase in admissions of older adults, but without improvement in 1-year mortality rates in a study from Israel [6].

Nonetheless, there may be some benefit for older adults to be admitted to ICUs. One report found that up to 50% of patients discharged from the hospital were still alive at 2 years [11]. Another study concluded that despite the fact that older adults have more intensive care unit refusals than younger patients and have higher mortality when admitted, the mortality reduction for older adults that are admitted to the ICU is greater than younger patients [12].

An alternative to a randomized control study is to make use of the existing differences between countries and health systems in the availability of ICU beds. The number of older adults treated in ICUs differs between countries, approximately in proportion to the available ICU beds [13]. This provides a setting for a natural experiment that can be utilized to evaluate the potential benefits or harms associated with increased ICU utilization in this sub-population. Results of such a study can facilitate discussion with patients and families based on accurate data about the true value of opting for management in intensive care units across different countries.

We aimed to assess if a non-restrictive approach to ICU admissions among eligible older adults improves in-hospital and long-term survival. We hypothesized that this approach would not increase long-term survival.

## Methods

### Study design, population, and outcomes

This is a retrospective cohort study that included patients aged 80 years or older who were admitted to each of the three participating hospitals—Soroka University Medical Center (SUMC) in Israel, Sir Charles Gairdner Hospital (SCGH) in Australia, and Beth Israel Deaconess Medical Center (BIDMC) in Boston, United States of America (USA). The three hospitals are all major tertiary level, academic, referral hospitals in their cities. Admissions in Israel and Australia were analyzed between the years 2006 and 2015 and in Boston between the years 2009–2015. These patients were sub-grouped into those who were admitted to an ICU and those who were admitted to a general ward. Only the first admission of every patient within the defined period of the study was analyzed. Within the index admission, only the first department and the first ICU admission were included in the analysis. Readmissions were considered as admissions occurring 6 months or less after discharge.

Exclusion criteria included: hospitalization for less than 24 h; admission for psychiatric conditions; admission for acute coronary syndrome as a primary diagnosis; advanced directives of CMO (comfort measures only) on day one of ICU admission; cardiac surgery patients (as elective patients) and absence of laboratory data on the admission.

The primary outcomes were in-hospital mortality and all-cause mortality at 6, 12, 18, and 24 months. Secondary outcomes were incidence of all ICU admissions among this age group in each medical center; ICU and ward mortality rates; ICU and hospital length of stay; and home discharge rates.

### Clinical definitions and data sources

We used the Charlson’s comorbidity index to quantify the severity of comorbidities and identified them by ICD-9 and ICD-10 codes during the hospitalizations and by data obtained from community clinics [14]. The Laboratory‐Based Acute Physiology Score (LAPS) is a validated score that predicts in-hospital mortality in various primary diagnoses based on laboratory data only [15]. The score ranges between a minimum of 0 and a theoretical maximum of 256. For the LAPS calculation, we included the first lab results of every test in the first admission.

The Australian data were linked using data from the hospital admission dataset, the state mortality dataset (with follow-up of 2 years after discharge), and the pathology provider.

Due to technical difficulties, we could not obtain long-term mortality data of the BIDMC cohort. Thus, for comparison on long-term mortality rates, we used the data of the Israeli and Australian cohorts and compared it to the ‘Medical Information Mart for Intensive Care’ (MIMIC-III) database between 2009 and 2012 [16]. This comparison is valid since MIMIC-III data are derived only from BIDMC, taken from years included in our study, and from the same ICUs, and therefore including an unbiased part of the same cohort.

### ICU capacities and admission policies

In all countries, ICU beds were calculated only if they are a part of a designated ICU unit with a team that is specialized in intensive care.

At SUMC, Beer Sheva, Israel, there are 827 adult beds and 24 ICU beds, respectively (ICU beds are 2.9% of all hospital beds, a ratio of 34:1). There are 16 general ICU beds and 8 medical ICU beds. This hospital is characterized by a highly restrictive triage policy. The admissions are at the discretion of the admitting intensive care specialist. Patients who are believed to have an irreversible clinical condition are mechanically ventilated in the internal medicine departments in most cases. ICU patients typically present with an acute need for mechanical ventilation or hemodynamic instability. SUMC is the only tertiary medical center in the city and serves as the tertiary center for a population of approximately 1 million people. It is the only hospital in the region of Southern Israel that provides cardiothoracic surgery, vascular surgery, and neurosurgery services.

At SCGH, Perth, Western Australia (WA), there are 600 adult and 23 adult ICU beds, respectively (ICU beds are 3.8% of all hospital beds, a ratio of 25:1). All the ICU beds are mixed with no differentiation between types of ICU. This ratio leads to a more restrictive ICU admission policy with ICU admission triaged on the basis of acuity, comorbidities, and prognosis. The admissions are at the discretion of the admitting intensive care specialist. No mechanical ventilation occurs outside of the single designated ICU areas in the hospital. There are 3 major referral ICUs in Perth, a city of 2 million people. SCGH is the only hospital in the state of WA providing neuro-interventional radiology services and liver transplantation. It also includes cardiothoracic surgery, vascular surgery, and neurosurgery services.

At BIDMC, Boston, USA, there are 621 adult and 77 ICU beds, respectively (ICU beds are 13% of all hospital beds, a ratio of 8:1). The breakup of ICU beds at BIDMC: 28 medical, 8 neurology, 18 trauma and surgical, 15 cardiovascular surgical, and 8 cardiac. This hospital is characterized by a liberal ICU admission policy wherein no broad-based exclusion criteria are implemented. When one of the ICUs is full (surgical-ICU, trauma-ICU, three medical-ICUs, cardiovascular-ICU, and cardiac-ICU), the patients are admitted in another suitable ICU or to the post-anesthesia care unit (PACU) as a “boarder”. This facilitates a policy of no refusal of ICU care for patients that are evaluated as requiring it. BIDMC is a tertiary level academic medical center serving Eastern Massachusetts and serves a catchment area of ~1 million people. Within the city of Boston (population of 4.5 million people) there are 5 other tertiary level academic medical centers and quite a few satellite hospitals within close proximity. Tertiary level centers in Boston provide similar services with few exceptions.

At SUMC and SCGH, ICU admissions are at the discretion of the admitting intensive care specialist and are “closed units” for the purposes of management, although teams are welcome to visit and provide their expertise and share in the care of the patient. In all centers, the medical insurance of the patient does not affect the ICU admission policy. Lawsuits are uncommon in intensive care in all countries.

### Statistical analysis

Demographics and baseline clinical characteristics are presented as mean (SD) or median (IQR) as appropriate. When appropriate, we made univariate comparisons using χ^2^-test for categorical variables and using one-way ANOVA or Kruskal–Wallis test for quantitative variables.

We used multivariable logistic regression to characterize the factors determining ICU admission in the American cohort. Variables with a significance of < 0.1 in the univariate analysis were inserted to the multivariable model. This model has a c-statistic of 0.8 demonstrating good discrimination. With the results of the logistic regression, we calculated a score for all patients, based on sex, age, Charlson score, LAPS, and primary admission diagnosis. The purpose of the score was to predict the probability of Israeli and Australian patients to be admitted to the ICU, based on the American model. All patients were divided into quintiles of probability to be admitted to the ICU. In every quintile, we compared the actual admission allocation and the in-hospital mortality rates between the different countries, both in the wards and in the ICUs.

Patients in the 5th quintile in the Israeli and Australian cohort, i.e., patients with the highest probability to be admitted to the ICU, according to the American model, were compared according to the primary and secondary outcomes. Mortality data were linked with the state death database in Australia, obtained from the MIMIC database for the USA, and from the state database in Israel. We calculated survival according to the Kaplan–Meier method with log-rank test.

A *p* value of 0.05 or less (two-sided) was considered statistically significant. Statistical analyses were performed with Stata, version 13.0 (StataCorp, Texas, USA) and SPSS, version 25 (IBM Corp).

## Results

### Study population

The study cohort included 62,866 patients aged 80 years or older who were admitted to any participating center during the study period. Israel, USA, and Australia comprised 17.2%, 57.1%, and 25.7% of the cohort, respectively.

The mean age was 85.93 ± 4.62 years, and 58.8% were women. The ICU admission rates differed substantially between Israel and Australia, with rates of 2.3% and 2.6%, respectively, and USA, with 22.5%. Duration of hospitalization was the longest in Australia, whereas ICU admission time was similar across centers (Table 1). Discharge destination also differed significantly between the centers, with 91.6% of the Israeli cohort discharged to their homes, compared to 42.9% and 52.0% in the American and Australian cohorts, respectively. In the American cohort, 35.1% were discharged to a long-term care/nursing home, compared to 9.2% in Australia. 19.7% of the Australian cohort were transferred to other hospitals.

**Table 1:** Characteristics of the patients at first admission

Variable	Israel (*n* = 10,847)	USA (*n* = 35,875)	Australia (*n* = 16,144)	All (*N* = 62,866)	*p* value
Age (years)					
Mean ± SD Median (IQR)	85.36 ± 4.42 84 (82–88)	86.19 ± 4.67 85 (82–89)	85.75 ± 4.6 85 (82–89)	85.93 ± 4.62 85 (82–89)	** < 0.001**
Female (*n*, %)	6410 (59.1%)	20,801 (57.98%)	9745 (60.36%)	36,956 (58.8%)	** < 0.001**
Hospital length of stay (days)					
Mean ± SD Median (IQR)	6.77 ± 8.35 4 (2–8)	6.09 ± 7.93 3 (5–7)	8.25 ± 9.16 5 (3–10)	6.76 ± 8.38 5 (3–8)	** < 0.001**
ICU admission rate	247 (2.28%)	8079 (22.52%)	417 (2.6%)	8743 (13.9%)	** < 0.001**
ICU length of stay (days)					
Mean ± SD Median (IQR)	3.79 ± 5.94 2 (1–4)	3.48 ± 5.04 2.05 (1.15–3.87)	3.92 ± 10.51 2 (1–4)	3.49 ± 5.07 2.04 (1.14–3.88)	0.34
LAPS score					
Mean ± SD Median (IQR)	28.98 ± 22.74 24 (11–42)	27.53 ± 19.81 24 (13–37)	22.51 ± 16.08 20 (11–31)	26.49 ± 19.63 23 (12–36)	** < 0.001**
In-hospital death (*n*, %)					
Ward ICU Total	725 (6.84%) 99 (40.08%) 824 (7.6%)	261 (0.94%) 1120 (13.86%) 1381 (3.85%)	689 (4.38%) 86 (20.62%) 775 (4.8%)	1675 (3.09%) 1305 (14.93%) 2980 (4.74%)	** < 0.001**
Discharged home	9931 (91.6%)	15,408 (42.9%)	8388 (52.0%)	33,727 (53.65%)	** < 0.001**
Charlson comorbidity index					
Mean ± SD Median (IQR)	5.37 ± 1.48 5 (4–6)	6.46 ± 2.24 6 (5–8)	4.93 ± 1.62 4 (4–5)	5.88 ± 2.09 5 (4–7)	** < 0.001**
Patients readmitted in any department (*n*, %)	3085 (28.4%)	13,338 (37.2%)	4934 (30.6%)	21,357 (34.0%)	** < 0.001**
Days to first readmission in any department (median, IQR)	36 (12–85)	31 (10–75)	30 (4–85)	31 (9–78.5)	** < 0.001**
Primary diagnosis (*n*, %)					
Abdominal pain	180 (1.66%)	155 (0.43%)	106 (0.66%)	441 (0.7%)	** < 0.001**
Cholecystitis/cholangitis	109 (1%)	246 (0.69%)	57 (0.35%)	412 (0.7%)	** < 0.001**
Acute kidney injury	107 (0.99%)	830 (2.31%)	167 (1.03%)	1104 (1.8%)	** < 0.001**
Acute pancreatitis	69 (0.64%)	161 (0.45%)	92 (0.57%)	322 (0.5%)	**0.03**
Acute respiratory failure	110 (1.01%)	218 (0.61%)	41 (0.25%)	369 (0.6%)	** < 0.001**
URTI	69 (0.64%)	53 (0.15%)	26 (0.16%)	148 (0.2%)	** < 0.001**
DVT PE	79 (0.73%)	329 (0.92%)	81 (0.5%)	489 (0.8%)	** < 0.001**
Anemia	142 (1.31%)	50 (0.14%)	119 (0.74%)	311 (0.5%)	** < 0.001**
Atrial fibrillation/flutter	247 (2.28%)	705 (1.97%)	288 (1.78%)	1240 (2.0%)	**0.02**
Cellulitis	105 (0.97%)	429 (1.2%)	190 (1.18%)	724 (1.2%)	0.14
Stroke and TIA	438 (4.04%)	909 (2.53%)	669 (4.14%)	2016 (3.2%)	** < 0.001**
Heart failure	257 (2.37%)	2222 (6.19%)	588 (3.64%)	3067 (4.9%)	** < 0.001**
Dizziness	85 (0.78%)	77 (0.21%)	70 (0.43%)	232 (0.4%)	** < 0.001**
Fever	589 (5.43%)	89 (0.25%)	20 (0.12%)	698 (1.1%)	** < 0.001**
Femoral fracture	564 (5.2%)	1030 (2.87%)	1485 (9.2%)	3079 (4.9%)	** < 0.001**
Hyponatremia	110 (1.01%)	171 (0.48%)	62 (0.38%)	343 (0.5%)	** < 0.001**
COPD	144 (1.33%)	277 (0.77%)	375 (2.3%)	796 (1.3%)	** < 0.001**
Dyspnea	505 (4.66%)	104 (0.29%)	30 (0.19%)	639 (1.0%)	** < 0.001**
Pneumonia	610 (5.62%)	1208 (3.37%)	680 (4.21%)	2498 (4.0%)	** < 0.001**
Syncope	304 (2.8%)	421 (1.17%)	245 (1.52%)	970 (1.5%)	** < 0.001**
Sepsis	411 (3.79%)	1433 (3.99%)	169 (1.05%)	2013 (3.2%)	** < 0.001**
Urinary tract infection	505 (4.66%)	1026 (2.86%)	410 (2.54%)	1941 (3.1%)	** < 0.001**

Bold value indicates significance *p* value < 0.05

*LAPS* laboratory-based Acute Physiology Score, *ICU* intensive care unit, *URTI* upper respiratory tract infection, *DVT PE* deep vein thrombosis and pulmonary embolism, *TIA* transient ischemic attack, *COPD* chronic obstructive pulmonary disease

Re-admission rate was highest among the American cohort (Table 1). The characteristics of patients with the highest probability of ICU admissions are described in Additional file 1: Table S1.

### Characteristics of ICU admission

The variables associated with higher probability of ICU admission in the American cohort were: younger age, male sex, higher LAPS scores, lower Charlson comorbidity index score, and the four following primary diagnoses—acute respiratory failure, sepsis, stroke or transient ischemic attack, and deep vein thrombosis or pulmonary embolism (Table 2). Detailed characteristics of patient admitted to the ICU compared to those who were not admitted are described in Additional file 1: Table S2. In the model predicting ICU admission probability, patients in the 5th quintile had a probability of 43–100% to be admitted to an ICU in USA. Characteristics of patients from the 5th quintile are described in Additional file 1: Tables S1 and S3. The actual ICU allocation of the patients in the fifth quintile was 67.6% in USA, 22.1% in Australia, and 6.0% in Israel (Table 3).

**Table 2:** Multivariable logistic regression model for ICU admission in the American cohort^a^

Variable	Odds ratio (95% CI)	*P* value	Coefficient
Female	0.94 (0.89–0.99)	0.046	− 0.059
Age			
90 and above 86–89 83–85 80–82	0.82 (0.75–0.89) 0.85 (0.79–0.92) 0.96 (0.88–1.04) 1	< 0.001 < 0.001 0.27 Reference	− 0.202 − 0.161 − 0.044 Reference
LAPS score			
38 and higher 24–37 15–23 6–14 0–5	14.65 (12.88–16.65) 3.43 (3.01–3.91) 2.03 (1.77–2.32) 1.35 (1.16–1.58) 1	< 0.001 < 0.001 < 0.001 < 0.001 Reference	2.684 1.234 0.707 0.304 Reference
Charlson comorbidity index			
7 and higher 6 0–5	0.92 (0.85–1.002) 0.86 (0.8–0.92) 1	0.05 < 0.001 Reference	0.079− − 0.151 Reference
Primary diagnosis^b^			
Acute respiratory failure	21.61 (12.53–37.25)	< 0.001	3.073
Sepsis	4.87 (4.27–5.55)	< 0.001	1.583
Stroke and TIA	2.25 (1.91–2.64)	< 0.001	0.81
DVT PE	1.45 (1.11–1.91)	0.01	0.374
Acute pancreatitis	0.88 (0.59–1.32)	0.55	− 0.123
Pneumonia	0.85 (0.73–0.99)	0.03	− 0.165
Cholecystitis/cholangitis	0.81 (0.6–1.11)	0.2	− 0.205
Atrial fibrillation/flutter	0.73 (0.58–0.92)	0.01	− 0.313
Heart failure	0.72 (0.64–0.81)	< 0.001	− 0.332
Femoral fracture	0.55 (0.45–0.66)	< 0.001	− 0.602
Dyspnea	0.21 (0.08–0.53)	< 0.001	− 1.557
Fever	0.17 (0.06–0.47)	< 0.001	− 1.779
UTI	0.17 (0.13–0.23)	< 0.001	− 1.756
Syncope	0.09 (0.04–0.18)	< 0.001	− 2.403

*LAPS* laboratory-based Acute Physiology Score, *ICU* intensive care unit, *URTI* upper respiratory tract infection, *DVT PE* deep vein thrombosis and pulmonary embolism, *TIA* transient ischemic attack, *COPD* chronic obstructive pulmonary disease, *UTI *urinary tract infection

^a^The model has c-statistic of 0.8, demonstrating good discrimination

^b^Diagnoses that were included in the model but do not appear in the table: abdominal pain, URTI, anemia, cellulitis, dizziness, hyponatremia, COPD

**Table 3:** Admission allocation (ICU vs. general ward) and in-hospital mortality rates according to different quintiles in every country

Country/quintile (predicted probability)	Admission allocation		1 (0–4%)	2 (5–8%)	3 (9–16%)	4 (17–42%)	5 (43–100%)	Total
United States, n (% of predicted probability)	Ward (% of entire quintile)	Actual Admission	2734 (97.61%)	5653 (92.29%)	9923 (88.39%)	7811 (73.97%)	1675 (32.44%)	27,796
		In-hospital death	5 (0.18%)	15 (0.27%)	51 (0.51%)	98 (1.25%)	92 (5.49%)	261
	ICU (% of entire quintile)	Actual Admission	67 (2.39%)	472 (7.71%)	1303 (11.61%)	2749 (26.03%)	3488 (67.56%)	8079
		In-hospital death	0 (0%)	16 (3.39%)	70 (5.37%)	264 (9.6%)	770 (22.08%)	1120
	Total (% of entire cohort)		2801 (7.81%)	6125 (17.07%)	11,226 (31.29%)	10,560 (29.44%)	5163 (14.39%)	35,875
Australia, n (% of predicted probability)	Ward (% of entire quintile)	Actual Admission	1473 (99.33%)	3079 (98.88%)	5507 (98.41%)	4700 (97.41%)	968 (85.97%)	15,727
		In-hospital death	20 (1.36%)	64 (2.08%)	172 (3.12%)	294 (6.26%)	139 (14.36%)	689
	ICU (% of entire quintile)	Actual Admission	10 (0.67%)	35 (1.12%)	89 (1.59%)	125 (2.59%)	158 (14.03%)	417
		In-hospital death	0 (0%)	6 (17.14%)	13 (14.61%)	20 (16%)	47 (29.75%)	86
	Total (% of entire cohort)		1483 (9.19%)	3114 (19.29%)	5596 (34.66%)	4825 (29.89%)	1126 (6.97%)	16,144
Israel, n (% of predicted probability)	Ward (% of entire quintile)	Actual admission	1682 (99.47%)	1897 (99.11%)	2715 (98.8%)	2559 (97.08%)	1747 (94.03%)	10,600
		In-hospital death	24 (1.43%)	24 (1.27%)	57 (2.1%)	147 (5.74%)	473 (27.07%)	725
	ICU (% of entire quintile)	Actual admission	9 (0.53%)	17 (0.89%)	33 (1.2%)	77 (2.92%)	111 (5.97%)	247
		In-hospital death	2 (22.22%)	4 (23.53%)	10 (30.3%)	25 (32.47%)	58 (52.25%)	99
	Total (% of entire cohort)		1691 (15.59%)	1914 (17.65%)	2748 (25.33%)	2636 (24.3%)	1858 (17.13%)	10,847
*p* value (difference between countries in ICU admissions)			< 0.001	< 0.001	< 0.001	< 0.001	< 0.001	< 0.001

Quintiles represent predicted probabilities for admission in an intensive care unit according to the American cohort (see Table 2)

### In-hospital and long-term mortality

In-hospital death rates were 7.6%, 4.8%, and 3.9% in Israel, Australia, and USA, respectively (Table 1). In the 5th quintile of the patients with the highest probability to be admitted to USA ICU, in-hospital death rates were 22.1% in Boston, 29.8% in Australia, and 52.3% in Israel (Table 3). The median initial Sequential Organ Failure Assessment (SOFA) score for patients admitted to the ICU in BIDMC was 4, compared to 8 in the Israeli ICU (Additional file 1: Table S2). The mortality rates of patients admitted to the ICU were 13.9% and 40.1% in USA and Israel, respectively, resulting in a standardized mortality ratio (SMR) of 0.69 and 1.2, according to the predicted mortality based on SOFA scores [17].

**Fig. 1:**
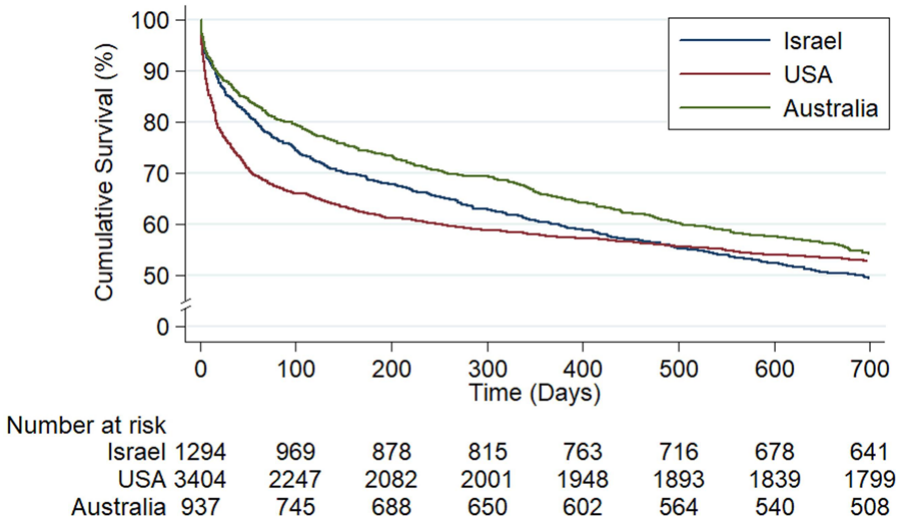
Kaplan–Meier curve comparing all-cause mortality of hospital survivors among Israeli and Australian patients in the 5th quintile (see Additional file 1: Table S5) and American patients from the MIMIC database

Figure 1 and Additional file 1: Table S5 show mortality data of patients with the highest probability of ICU admissions in the Israeli and Australian cohorts, compared to the MIMIC-III cohort of BIDMC. After 6 and 12 months the survival rates were 62% and 58%, 74% and 66%, and 69% and 60% in USA, Australia, and Israel, respectively (*p* value < 0.001 for both periods, log-rank test). At 24 months, the survival rates in the USA and Australia were 53%, while in Israel 48% (*p* value 0.06).

## Discussion

To the best of our knowledge, this is the first comparison of in-hospital and short- and long-term mortality of older adults (≥ 80 years) from three different developed countries across three continents. We found that older adults at a medical center with high ICU capacity (USA) were admitted to the ICU ten times more than in Israel and Australia, which have much lower ICU capacities. In-hospital deaths were negatively associated with ICU admission rate, lowest in the USA and highest in Israel.

The predicted in-hospital mortality for LAPS scores of 20–29 is ~ 3% [15]. The mean LAPS score in our entire cohort was 26 and the overall mortality was 4.7%, higher than predicted by the LAPS score, with significant differences between the countries as described in Table 1. The higher-than-expected mortality rate was observed also in the ICUs, where the mean LAPS score was 43.9 (predicted in-hospital mortality is ~ 7%) and the overall mortality was 14.9%. This difference between the observed and expected mortality can be explained by the difference between our study population and the population which was used to create the LAPS score. The LAPS score was created from a general population that included also younger patients with less comorbidities, as opposed to our population, and the score does not consider comorbidities at all, only laboratory results at admission. When considering only in-ICU mortality, the difference between the initial SOFA scores of Israel and USA reflects the difference in the severity of the patients and thus the difference in the mortality data. In contrast to its lowest in-hospital mortality rate, the 1-year mortality rate was highest in the American cohort when comparing patients admitted to ICUs at USA with Israeli and Australian patients that had the highest probability for ICU admission. The time to readmission was shorter in the American and Australian cohorts. This supports the hypothesis that although ICU admission reduces in-hospital mortality it does not significantly improve long-term outcomes such as readmissions and mortality. This finding is consistent with another previous study [18]. Another contributing factor for this finding is the higher Charlson scores in the American cohort compared to the other two which increases the likelihood of mortality and shortens readmission times. A large randomized controlled trial showed that a program to promote ICU admission of older adults significantly increased ICU admissions, but did not reduce 6 months mortality [19]. On the contrary, a large international study concluded that although older adults have more intensive care unit refusals than younger patients and suffer from higher mortality rates when admitted to an ICU, their actual survival benefit when they enter the ICU is greater than younger patients [12]. Whereas readmissions to the hospital occurred in about a third of the cases in all countries, readmission of patients hospitalized in the ICU on their index admission and then again in the ICU within 6 months were rare in Israel and much more common in Australia and in the USA (Additional file 1: Table S2). This also reflects the different ICU admission policies between the hospitals.

The rates of patients that were discharged home were also significantly different between the countries. This shows that increasing ICU beds capacity, which is expected to lead to increased survival rates, must be accompanied by appropriate long-term facilities that can handle the increasing numbers of older adults surviving hospital admissions. The rates of patients discharged home in the American and Australian cohorts was similar to other American and German cohorts [20, 21].

Long-term mortality may be affected by the underlying health of the population in that country. However, for these three countries, the life expectancy at age 80 is very similar (in Australia 9.87 years, in USA 9.6, and in Israel 9.66 years) [22, 23].

For patients with advanced malignancy, therapies that prolong life expectancy in weeks or months are considered substantial and many resources are invested in them [24]. It cannot be justified to withhold ICU admission for all patients above a certain age. At times of scarcity, however, it may be justified to prioritize the younger patients, in order to maximize the benefits for the largest number of people [25].

Augmentation of ICU capacity may allow admission of older adults and in-hospital mortality benefits may accrue. However, there is still the question of whether the addition of beds always means that more lives will be saved or whether there is a point at which no additional mortality benefit will be gained. With an abundance of ICU beds may come the possibility of increasing harm in the forms of unnecessary costs, poor quality of deaths (i.e., excessively intensive), poor quality of life after ICU discharge, and iatrogenic complications [26]. The identification of those very old, who survived a long time after an ICU admission, with an acceptable quality of life, compared to those that did not should be the focus of future research. The ethical question: how much money, personnel, ICU beds, and ICU units should we invest to save a life, even in the short term, is a relevant question for ethicists, health care personnel, health care administrators, and society. The answer to these questions might also vary substantially between the countries in our study and between other developed countries with distinct socio-cultural beliefs. This is a question not too far away from challenging us as in years to come the proportion of older adults continues to increase.

Our study has some limitations. We did not assess frailty and quality of life to any of our patients admitted to the hospital or who survived the ICU admission. Being discharged alive from the ICU is not necessarily the main preference of our patients and families [27]. We believe that the assessment of functional status, well-being, and quality of life in this population should be the focus of future studies. We did not assess any of the costs related to ICU admissions, ward admissions, long-term facilities on health care, medical centers, community, and families. Differences identified between the countries may reflect the population underlying characteristics, either in terms of health, different outpatient care structure, different age composition of the population, life expectancy, or post-hospitalization care. Measurable characteristics are similar for the different populations, but unmeasured confounders cannot entirely be ruled out. Our data are not able to provide a reason for the large difference in number of patients between the countries despite similar number of hospital beds overall. In addition, only one hospital represents each country in the study and none of the studied hospitals can be a representative of the entire country. Nevertheless, it should be emphasized that the main comparison is the ICU bed saturation (ICU beds to hospital beds ratio), which is similar in other hospitals within each country. The long-term mortality was not available to us from the American cohort. Only in-hospital mortality was documented. We used MIMIC III data, extracted from the same hospital (BIDMC), age, ICUs, and included some years of study. We believe that these data reflect accurately the long-term survival of the older adults from our American cohort.

## Conclusions

Comparing three large academic medical centers from three different countries and continents, higher ICU bed capacity and more liberal ICU admission policies are associated with higher in-hospital survival of older adults, but long-term survival (6–24 months) is similar, and number of readmissions is higher. The strategies for allocation of ICU beds for older adults remain an area of further research.

## Supplementary Information

**Additional file 1: Table S1.** Characteristics of the 5th quintile from Table 3. **Table S2.** Characteristics of The Patients at First Admission, Comparing ICU Admitted Patients to Ward Admitted Patients. **Table S3.** Characteristics of the patients admitted to the ICU in the 5th quintile from Table S1. **Table S4.** Characteristics of the patients not admitted to the ICU in the 5th quintile from. **Table S5.** Kaplan–Meier mean estimates for 4 time points.

## Data Availability

The data used in the analysis of this study are not publicly available due to the requirements of the IRB Committee, can be considered by the IRB committee and data custodians through request via the corresponding author.

## Abbreviations

ICU: Intensive care unit
SUMC: Soroka University Medical Center
SCGH: Sir Charles Gairdner Hospital
BIDMC: Beth Israel Deaconess Medical Center
USA: United States of America
CMO: Comfort measures only
LAPS: Laboratory‐Based Acute Physiology Score
MIMIC-III: Medical information mart for intensive care-III
