# Supplementary material for: Intensive care unit capacity and mortality in older adults: a three nations retrospective observational cohort study
Source: Ann Intensive Care. 2022 Mar 4;12:20. doi: 10.1186/s13613-022-00994-x (PMC8897522; doi:10.1186/s13613-022-00994-x)
Supplement: Supplementary file 1 — Additional file 1: Table S1. Characteristics of the 5th quintile from Table 3. Table S2. Characteristics of The Patients at First Admission, Comparing ICU Admitted Patients to Ward Admitted Patients. Table S3. Characteristics of the patients admitted to the ICU in the 5th quintile from Table S1. Table S4. Characteristics of the patients not admitted to the ICU in the 5th quintile from. Table S5. Kaplan–Meier mean estimates for 4 time points. [file 13613_2022_994_MOESM1_ESM.docx]

**Supplementary Tables**

**Supplementary Table 1** – Characteristics of the 5^th^ quintile from Table 3

| **Variable** | **Israel**  **(n=1,858)** | **USA**  **(n=5,163)** | **Australia**  **(n=1,126)** |
| --- | --- | --- | --- |
| **Age (years, mean ± SD)** | 85.07 ± 4.4 | 85.62 ± 4.45 | 84.74 ± 4.03 |
| **Female (n, %)** | 1011 (54.41%) | 2614 (50.63%) | 543 (48.22%) |
| **Hospital Length of stay (days, median, IQR)** | 6 (3-11) | 8 (5-12) | 7 (3-12) |
| **LAPS Score^1^ (mean ± SD)** | 63.44 ± 19.02 | 62.32 ± 17.31 | 58.86 ± 16.71 |
| **Charlson Comorbidity Index (mean ± SD)** | 5.7 ± 1.55 | 7.03 ± 2.18 | 5.24 ± 1.56 |
| **Patients readmitted (n, %)** | 729 (39.24%) | 2157 (41.78%) | 565 (50.18%) |
| **Days to First Readmission (median, IQR)** | 67 (15-256) | 38 (10-136.5) | 108 (8-413) |
| **Primary diagnosis (n, %)** |  |  |  |
| **Cholecystitis/cholangitis** | 31 (1.67%) | 56 (1.08%) | 11 (0.98%) |
| **Acute kidney injury** | 28 (1.51%) | 54 (1.05%) | 14 (1.24%) |
| **Acute pancreatitis** | 3 (0.16%) | 25 (0.48%) | 12 (1.07%) |
| **Acute respiratory failure** | 109 (5.87%) | 218 (4.22%) | 41 (3.64%) |
| **DVT PE** | 18 (0.97%) | 46 (0.89%) | 10 (0.89%) |
| **Atrial fibrillation/flutter** | 19 (1.02%) | 27 (0.52%) | 8 (0.71%) |
| **Stroke and TIA** | 69 (3.71%) | 135 (2.61%) | 55 (4.88%) |
| **Heart failure** | 66 (3.55%) | 230 (4.45%) | 35 (3.11%) |
| **Fever** | 24 (1.29%) | 0 (0%) | 0 (0%) |
| **Femoral fracture** | 21 (1.13%) | 76 (1.47%) | 12 (1.07%) |
| **Chronic obstructive pulmonary disease** | 56 (3.01%) | 35 (0.68%) | 0 (0%) |
| **Pneumonia** | 180 (9.69%) | 165 (3.2%) | 96 (8.53%) |
| **Sepsis** | 373 (20.08%) | 1255 (24.31%) | 122 (10.83%) |
| **Urinary tract infection** | 33 (1.78%) | 2 (0.04%) | 5 (0.44%) |

LAPS - Laboratory-based Acute Physiology Score; DVT PE – Deep vein thrombosis and pulmonary embolism; TIA – Transient ischemic attack.

**Supplementary Table 2** – Characteristics of The Patients at First Admission, Comparing ICU Admitted Patients to Ward Admitted Patients.

| **Variable** | **Israel**  **(n=10847)** | | **USA**  **(n=35875)** | | **Australia**  **(n=16144)** | | **All**  **(N=62866)** | |
| --- | --- | --- | --- | --- | --- | --- | --- | --- |
|  | **ICU**  **(n=247)** | **Ward (n=10600)** | **ICU**  **(n=8079)** | **Ward (n=27796)** | **ICU**  **(n=417)** | **Ward (n=15727)** | **ICU**  **(n=8743)** | **Ward (n=54123)** |
| **Age (years)**  **Mean ± SD**  **Median (IQR)** | 84.8 ± 4.2  84 (82-87) | 85.4 ± 4.4  84 (82-88) | 86.0 ± 4.6  85 (82-89) | 86.3 ± 4.7  86 (82-89) | 83.7 ± 3.4  83 (81-85) | 85.8 ± 4.6  85 (82-89) | 85.8 ± 4.6  85 (82-89) | 86.0 ± 4.6  85 (82-89) |
| **Female (n, %)** | 143 (57.9%) | 6267 (59.1%) | 4347 (53.8%) | 16454 (59.2%) | 178 (42.7%) | 9567 (60.8%) | 4668 (53.4%) | 32288 (59.7%) |
| **Hospital Length of stay (days)**  **Mean ± SD**  **Median (IQR)** | 20.1 ± 20.8  13 (6-25) | 6.5 ± 7.6  4 (2-7) | 9.3 ± 14.7  7 (5-11) | 5.2 ± 3.8  4 (3-6) | 16.0 ± 17.1  11 (6-21) | 8.0 ± 8.8  5 (3-10) | 9.9 ± 15.2  8 (5-12) | 6.3 ± 6.5  4 (3-7) |
| **LAPS Score**  **Mean ± SD**  **Median (IQR)** | 48.0 ± 26.0  46 (28-66) | 28.5 ± 22.5  24 (11-41) | 43.9 ± 23.7  42 (26-59) | 22.8 ± 15.6  20 (11-31) | 40.8 ± 26.5  36 (18-59) | 22.0 ± 15.4  19 (11-31) | 43.9 ± 23.9  41 (25-59) | 23.7 ± 17.3  21 (11-33) |
| **Initial SOFA Score, median (IQR)** | 8 (5-10) | - | 4 (2-6) | - | -* | - | - | - |
| **Discharged Home** | 140 (56.7%) | 9791 (92.4%) | 2284 (28.3%) | 13124 (47.2%) | 123 (29.5%) | 8265 (52.6%) | 2547 (29.1%) | 31180 (57.6%) |
| **Discharged to Long-term care/Nursing home** | 7 (2.8%) | 47 (0.4%) | 3849 (47.6%) | 8993 (32.3%) | 6 (1.4%) | 1599 (10.2%) | 3862 (44.2%) | 10639 (19.7%) |
| **Charlson Comorbidity Index**  **Mean ± SD**  **Median (IQR)** | 5.6 ± 1.6  5 (4-7) | 5.4 ± 1.5  5 (4-6) | 6.8 ± 2.3  6 (5-8) | 6.4 ± 2.2  6 (5-7) | 5.1 ± 1.6  4 (4-6) | 4.9 ± 1.6  4 (4-5) | 6.7 ± 2.3  6 (5-8) | 5.8 ± 2.0  5 (4-7) |
| **Patients readmitted in any department (n, %)** | 43 (17.4%) | 3042 (28.7%) | 2496 (30.9%) | 10842 (39.0%) | 105 (25.2%) | 4829 (30.7%) | 2644 (30.2%) | 18713 (34.6%) |
| **Number of readmissions (Median, IQR)** | 0 (0-1) | 0 (0-2) | 2 (1-3) | 2 (1-4) | 0 (0-2) | 1 (0-2) | 1 (1-3) | 1 (0-3) |
| **Days to First Readmission (median, IQR)** | 18 (6-38) | 36 (12-86) | 22 (8-62) | 33 (11-78) | 20 (1.5-76) | 30 (5-85) | 22 (8-63) | 33 (10-81) |
| **ICU readmission rates** | 1 (0.4%) | – | 767 (9.5%) | – | 21 (5.0%) | – | 789 (9.0%) | – |
| **Primary diagnosis (n, %)** |  | | | | | | | |
| **Abdominal pain** | 4 (1.6%) | 176 (1.7%) | 2 (0%) | 153 (0.6%) | 0 (0%) | 106 (0.7%) | 6 (0.1%) | 435 (0.8%) |
| **Cholecystitis/cholangitis** | 11 (4.5%) | 98 (0.9%) | 64 (0.8%) | 182 (0.7%) | 5 (1.2%) | 52 (0.3%) | 80 (0.9%) | 332 (0.6%) |
| **Acute kidney injury** | 5 (2%) | 102 (1%) | 116 (1.4%) | 714 (2.6%) | 4 (1%) | 163 (1%) | 125 (1.4%) | 979 (1.8%) |
| **Acute pancreatitis** | 7 (2.8%) | 62 (0.6%) | 37 (0.5%) | 124 (0.4%) | 2 (0.5%) | 90 (0.6%) | 46 (0.5%) | 276 (0.5%) |
| **Acute respiratory failure** | 0 (0%) | 110 (1%) | 203 (2.5%) | 15 (0.1%) | 4 (1%) | 37 (0.2%) | 207 (2.4%) | 162 (0.3%) |
| **URTI** | 7 (2.8%) | 62 (0.6%) | 1 (0%) | 52 (0.2%) | 0 (0%) | 26 (0.2%) | 8 (0.1%) | 140 (0.3%) |
| **DVT PE** | 3 (1.2%) | 76 (0.7%) | 84 (1%) | 245 (0.9%) | 1 (0.2%) | 80 (0.5%) | 88 (1%) | 401 (0.7%) |
| **Anemia** | 1 (0.4%) | 141 (1.3%) | 4 (0%) | 46 (0.2%) | 1 (0.2%) | 118 (0.8%) | 6 (0.1%) | 305 (0.6%) |
| **Atrial fibrillation/flutter** | 1 (0.4%) | 246 (2.3%) | 102 (1.3%) | 603 (2.2%) | 9 (2.2%) | 279 (1.8%) | 112 (1.3%) | 1128 (2.1%) |
| **Cellulitis** | 1 (0.4%) | 104 (1%) | 14 (0.2%) | 415 (1.5%) | 0 (0%) | 190 (1.2%) | 15 (0.2%) | 709 (1.3%) |
| **Stroke and TIA** | 17 (6.9%) | 421 (4%) | 257 (3.2%) | 652 (2.3%) | 12 (2.9%) | 657 (4.2%) | 286 (3.3%) | 1730 (3.2%) |
| **Heart failure** | 4 (1.6%) | 253 (2.4%) | 451 (5.6%) | 1771 (6.4%) | 10 (2.4%) | 578 (3.7%) | 465 (5.3%) | 2602 (4.8%) |
| **Dizziness** | 0 (0%) | 85 (0.8%) | 1 (0%) | 76 (0.3%) | 1 (0.2%) | 69 (0.4%) | 2 (0%) | 230 (0.4%) |
| **Fever** | 3 (1.2%) | 586 (5.5%) | 4 (0%) | 85 (0.3%) | 0 (0%) | 20 (0.1%) | 7 (0.1%) | 691 (1.3%) |
| **Femoral fracture** | 39 (15.8%) | 525 (5%) | 153 (1.9%) | 877 (3.2%) | 19 (4.6%) | 1466 (9.3%) | 211 (2.4%) | 2868 (5.3%) |
| **Hyponatremia** | 0 (0%) | 110 (1%) | 25 (0.3%) | 146 (0.5%) | 2 (0.5%) | 60 (0.4%) | 27 (0.3%) | 316 (0.6%) |
| **COPD** | 1 (0.4%) | 143 (1.3%) | 49 (0.6%) | 228 (0.8%) | 0 (0%) | 0 (0%) | 50 (0.6%) | 371 (1%) |
| **Dyspnea** | 6 (2.4%) | 499 (4.7%) | 5 (0.1%) | 99 (0.4%) | 0 (0%) | 30 (0.2%) | 11 (0.1%) | 628 (1.2%) |
| **Pneumonia** | 12 (4.9%) | 598 (5.6%) | 276 (3.4%) | 932 (3.4%) | 20 (4.8%) | 660 (4.2%) | 308 (3.5%) | 2190 (4%) |
| **Syncope** | 0 (0%) | 304 (2.9%) | 8 (0.1%) | 413 (1.5%) | 4 (1%) | 241 (1.5%) | 12 (0.1%) | 958 (1.8%) |
| **Sepsis** | 38 (15.4%) | 373 (3.5%) | 1036 (12.8%) | 397 (1.4%) | 16 (3.8%) | 153 (1%) | 1090 (12.5%) | 923 (1.7%) |
| **Urinary tract infection** | 3 (1.2%) | 502 (4.7%) | 47 (0.6%) | 979 (3.5%) | 2 (0.5%) | 408 (2.6%) | 52 (0.6%) | 1889 (3.5%) |

* SOFA score data were not available for the Australian cohort.

LAPS – Laboratory-based Acute Physiology Score; SOFA – Sequential Organ Failure Assessment; ICU – Intensive care unit; URTI – Upper respiratory tract infection; DVT PE – Deep vein thrombosis and pulmonary embolism; TIA – Transient ischemic attack; COPD – Chronic obstructive pulmonary disease.

**Supplementary Table 3** – Characteristics of the patients admitted to the ICU in the 5^th^ quintile from Table 3

| **Variable** | **Israel**  **(n=111)** | **USA**  **(n=3,488)** | **Australia**  **(n=158)** |
| --- | --- | --- | --- |
| **Age (years, mean ± SD)** | 84.59 ± 4.5 | 85.82 ± 4.5 | 83.38 ± 3.1 |
| **LAPS Score (mean ± SD)** | 68.24 ± 20.3 | 64.34 ± 18.0 | 68.47 ± 17.9 |
| **Charlson Comorbidity Index (mean ± SD)** | 5.88 ± 1.7 | 7.05 ± 2.4 | 5.18 ± 1.4 |
| **Mean probability for ICU admission* (% ± SD)** | 71.51 ± 1.7 | 69.46 ± 1.7 | 69.58 ± 1.7 |

* ICU admission in Boston, according to the model of Table 2.

ICU – Intensive care unit; LAPS - Laboratory-based Acute Physiology Score.

**Supplementary Table 4** – Characteristics of the patients not admitted to the ICU in the 5^th^ quintile from Table 3

| **Variable** | **Israel**  **(n=1,747)** | **USA**  **(n=1,675)** | **Australia**  **(n=968)** |
| --- | --- | --- | --- |
| **Age (years, mean ± SD)** | 85.62 ± 4.6 | 85.91 ± 4.5 | 85.42 ± 4.4 |
| **LAPS Score (mean ± SD)** | 63.13 ± 18.9 | 58.11 ± 14.8 | 57.29 ± 16.0 |
| **Charlson Comorbidity Index (mean ± SD)** | 5.62 ± 1.6 | 7.15 ± 2.4 | 5.26 ± 1.7 |
| **Mean probability for ICU admission* (% ± SD)** | 67.28 ± 1.6 | 59.07 ± 1.4 | 60.06 ± 1.5 |

* ICU admission in Boston, according to the model of Table 2.

ICU – Intensive care unit; LAPS - Laboratory-based Acute Physiology Score.

**Supplementary Table 5** – Kaplan-Meyer mean estimates for 4 time points.

| **Country/ KM estimate (Std. Error)** | **6 months** | **12 months** | **18 months** | **24 months** |
| --- | --- | --- | --- | --- |
| **Israel** | 0.69 (0.013) | 0.60 (0.013) | 0.54 (0.014) | 0.48 (0.014) |
| **USA** | 0.62 (0.008) | 0.58 (0.008) | 0.55 (0.009) | 0.53 (0.009) |
| **Australia** | 0.74 (0.013) | 0.66 (0.016) | 0.59 (0.016) | 0.53 (0.016) |
